# Genome-wide tracking of dCas9-methyltransferase footprints

**DOI:** 10.1038/s41467-017-02708-5

**Published:** 2018-02-09

**Authors:** Christina Galonska, Jocelyn Charlton, Alexandra L. Mattei, Julie Donaghey, Kendell Clement, Hongcang Gu, Arman W. Mohammad, Elena K. Stamenova, Davide Cacchiarelli, Sven Klages, Bernd Timmermann, Tobias Cantz, Hans R. Schöler, Andreas Gnirke, Michael J. Ziller, Alexander Meissner

**Affiliations:** 1 Department of Genome Regulation, Max Planck Institute for Molecular Genetics, 14195 Berlin, Germany; 2 Department of Stem Cell and Regenerative Biology, Harvard University, Cambridge, MA 02138 USA; 3 Broad Institute of MIT and Harvard, Cambridge, MA 02142 USA; 4 Translational Hepatology and Stem Cell Biology, Hannover Medical School, Hannover, 30625 Germany; 5 Department of Cell and Developmental Biology, Max Planck Institute for Molecular Biomedicine, Münster, 48149 Germany; 6 Department for Translational Psychiatry, Max Planck Institute of Psychiatry, Munich, 80804 Germany; 7 Present Address: Armenise-Harvard Laboratory of Integrative Genomics, Telethon Institute of Genetics and Medicine (TIGEM), Pozzuoli, 80078 Italy

## Abstract

In normal mammalian development cytosine methylation is essential and is directed to specific regions of the genome. Despite notable advances through mapping its genome-wide distribution, studying the direct contribution of DNA methylation to gene and genome regulation has been limited by the lack of tools for its precise manipulation. Thus, combining the targeting capability of the CRISPR–Cas9 system with an epigenetic modifier has attracted interest in the scientific community. In contrast to profiling the genome-wide cleavage of a nuclease competent Cas9, tracing the global activity of a dead Cas9 (dCas9) methyltransferase fusion protein is challenging within a highly methylated genome. Here, we report the generation and use of an engineered, methylation depleted but maintenance competent mouse ES cell line and find surprisingly ubiquitous nuclear activity of dCas9-methyltransferases. Subsequent experiments in human somatic cells refine these observations and point to an important difference between genetic and epigenetic editing tools that require unique experimental considerations.

## Abstract (web summary)

Catalytically inactive Cas9 fused to a methyltransferase has emerged as a promising epigenome modifying tool. Here the authors generate a methylation depleted but maintenance competent mouse ES cell line and find ubiquitous off-target activity.

## Introduction

DNA methylation is widespread among organisms, with the core enzymes that catalyze the methyl group transfer being conserved for more than a billion years across plants and animals^1–3^. Comparative genome-wide DNA methylation mapping has enhanced our understanding of the mammalian targets and dynamics of this modification^2,4–7^, but many important questions regarding its precise regulatory role remain unanswered. The complex multilayered mechanisms by which DNA methylation is regulated and mitotically maintained complicate its study and the absence of tools that enable targeted manipulation has limited progress further.

However, recent advances in the field of genome editing have raised hopes that these technical limitations may finally be overcome^8^. In particular, the CRISPR-Cas9 system for genome engineering has emerged as a powerful genomics toolbox due to its high targeting specificity and efficiency^9^. More recently, fusion of effector domains or proteins to the catalytically inactive (dead) dCas9 protein extended the potential applications to targeted epigenome editing^9–16^, including de novo methylation through dCas9-methyltransferase fusion proteins. However, several critical questions need to be explored before DNA methylation editing can be considered a reliable tool. It remains unclear what characteristics render a given locus susceptible to become ectopically methylated, i.e. how much does the transcriptional or chromatin state of a given target matter? Can canonically unmethylated regions be targeted and the methylation maintained in the absence of the inducer? For instance, recent studies suggest that directed methylation can alter target gene expression, although methylation is rapidly lost upon removal of the dCas9-effector^12,13,17^. How dependent is the dCas9-methyltransferase on the presence of the endogenous de novo machinery? Finally, how much off-target activity arises when the dCas9-methyltransferase complex is present in the nucleus near its substrate (all cytosines)?

As for the latter, previous studies show that the nuclease active Cas9 rarely cuts at off-target sites, despite widespread engagement as shown by genome-wide mapping^18^. However, chromatin immunoprecipitation (ChIP)-based approaches are not sensitive enough to detect transient or past interactions, which may be sufficient to induce lasting epigenetic alterations such as DNA methylation. Furthermore, high levels of DNA methylation and the presence of the endogenous de novo DNA methyltransferases (Dnmts) complicate any accurate evaluation of dCas9-methyltransferase activity in the nucleus^5,7^. Limited by these factors, current literature offers preliminary insights into the general applicability and on-target methylation efficiency of dCas9-fused methylation effectors yet lacks a general interpretation of global off-target activity. The same drawbacks have also restricted the precise measurement of seeding, spreading and maintenance of targeted DNA methylation. Here, we present a system to measure several of these parameters and explore the effects of dCas9-methyltransferases in pluripotent cells. We observe widespread off-target activity of dCas9-methyltransferases, which occurres independently of the presence of single guide RNAs (sgRNAs) and was also apparent across multiple somatic cell types. Our results therefore provide valuable insight into the utility of epigenome editing tools that should be considered in future experimental designs.

## Results

### Generation of an ES cell model to track de novo methylation

**Fig. 1:**
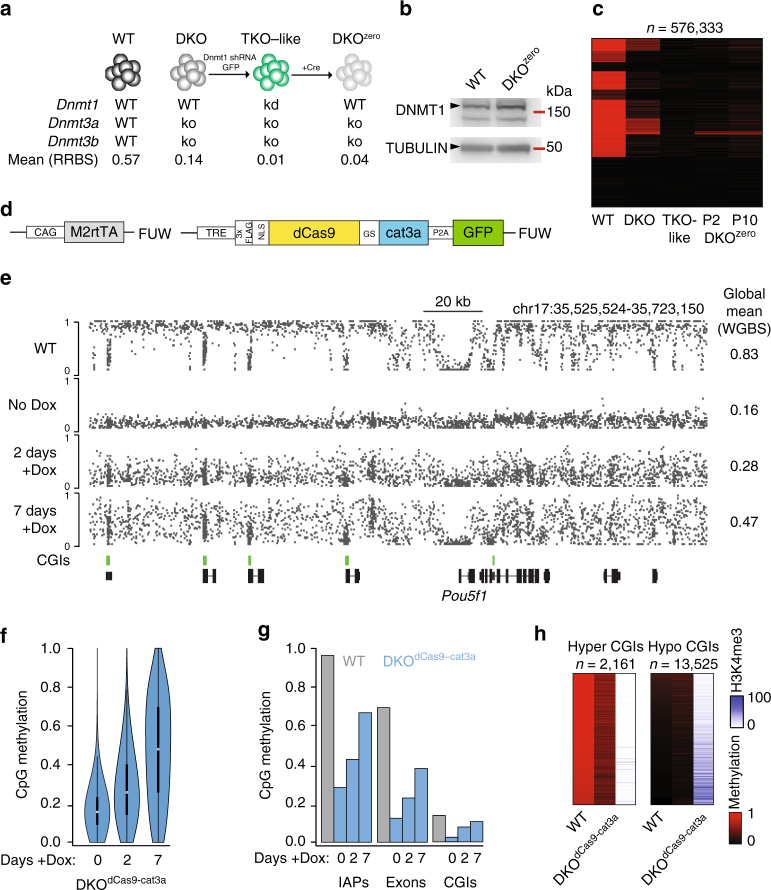
Characterization of dCas9-cat3a activity in a methylation depleted cell line. **a** Overview of the multi-step generation of the Dnmt3 (de novo) free, maintenance competent, methylation depleted DKO^zero^ ES cell line (see Methods section for details), WT = wild-type, kd = knock down, ko = knockout. Mean = mean methylation for matched CpGs measured by reduced representation bisulfite sequencing (RRBS). **b** Western blot of DNMT1 (183 kDa) in WT and DKO^zero^ cells. TUBULIN (50 kDa) serves as loading control. **c** Heatmap comparing global CpG methylation levels measured by RRBS in WT, DKO, TKO-like, early (P2) and late (P10) passage DKO^zero^ cells. *n* shows the number of shared CpGs (*n* = 576,333). **d** Simplified schematics showing the M2rtTA and dCas9-cat3a FUW-based lentiviral expression constructs. **e** Representative whole genome bisulfite sequencing (WGBS) tracks displaying CpG methylation in WT as well as reset DKO^dCas9-cat3a^ cells with 0, 2, or 7 days of Dox induction. Global mean CpG methylation levels are shown on the right. CpG islands (CGIs) are highlighted beneath in green. **f** Violin plots for matched CpGs in DKO^dCas9-cat3a^ with 0, 2, and 7 days of Dox induction without the presence of an sgRNA. White dots indicate median values. **g** Mean CpG methylation across selected features in WT and DKO^dCas9-cat3a^ cells with 0, 2, and 7 days of Dox induction. IAPs = intra-cisternal A-type particles (retrotransposons). **h** Heatmap for all hypermethylated CGIs (mean in WT > 0.8, left) and hypomethylated CGIs (mean in WT ≤ 0.2, right) in WT and DKO^dCas9-cat3a^ cells induced for 7 days with Dox. H3K4me3 enrichment for each CGI in WT ES cells is displayed

To systematically assess the global effects of dCas9-fused methyltransferases, we used previously established *Dnmt3a/b* double knockout (DKO) embryonic stem (ES) cells^19^ and transiently repressed the maintenance methyltransferase *Dnmt1* to deplete global methylation levels (as described previously^20^). This strategy allowed us to derive a new cell line (termed DKO^zero^) that has significantly reduced cytosine methylation (mean global CpG methylation measured by reduced representation bisulfite sequencing (RRBS)/ whole-genome bisulfite sequencing (WGBS): wild-type ES cells 0.57/0.83 and DKO^zero^: 0.04/0.04) but is able to maintain any emerging DNA methylation through the re-expression of endogenous *Dnmt1* (Fig. 1a, b, Supplementary Fig. 1a, b; Methods section). As a reference point, the global CpG methylation levels in the DKO^zero^ cell line are below what has been observed for wild-type ES cells grown in 2i/LIF conditions^21^ (Supplementary Fig. 1a). Continued passaging of the DKO^zero^ cells did not result in any notable gain of global DNA methylation, suggesting limited de novo activity of DNMT1 (Fig. 1c, Supplementary Fig. 1c). As a result, these DNA methylation depleted, maintenance competent ES cells allow us to precisely track the extent of newly added DNA methylation (without interference from the endogenous de novo enzymes) including past engagements of the catalytic domain with unmethylated cytosines that are subsequently propagated by DNMT1.

### dCas9–cat3a displays global nuclear activity

After we established our DKO^zero^ system, we generated a doxycycline (Dox) responsive construct that when co-introduced with the reverse trans-activator (M2rtTA) enables the inducible expression of dCas9–cat3a (dCas9 fused to the catalytic domain of Dnmt3a; Fig. 1d). After viral transduction and selection for double positive cells (M2rtTA plus the inducible construct), we derived a clonal cell line (DKO^dCas9–cat3a^) and assessed dCas9-cat3a induction by measuring GFP expression using fluorescence activated cell sorting (FACS). Based on this analysis we observed homogenous induction in the majority of cells (Supplementary Fig. 1d) with methylation analysis confirming no detectable leakiness in the absence of Dox (Supplementary Fig. 1e). However, we noticed to our surprise that the transient induction with Dox had already led to widespread gain of CpG methylation (Supplementary Fig. 1f, left), suggesting strong global activity by dCas9–cat3a. To measure the impact of dCas9–cat3a induction more precisely, we reset global methylation in the DKO^dCas9–cat3a^ line through another round of transient Dnmt1 depletion as well as transient culture in 2i/LIF plus Vitamin C, a combination that rapidly reduces global DNA methylation^22^. After again confirming the global depletion of DNA methylation (Supplementary Fig. 1f, right), we induced the reset DKO^dCas9–cat3a^ cell line in the absence of a sgRNA for 0 (uninduced control), 2 and 7 days and performed WGBS (Fig. 1e, f). This data not only confirmes our prior observation but also highlights how rapid and widespread the increase in DNA methylation occurs (mean CpG methylation uninduced: 0.16; 2 days: 0.28 and 7 days: 0.47). Upon closer inspection, we found that the gain of methylation is largely defined by the prior methylation state in wild-type ES cells. Specifically, genomic features that were highly methylated in wild-type ES cells, such as exons and repetitive elements, showed a notable gain upon Dox induction, as did regions of intermediate methylation (mean >0.2 and <0.8 in wild-type cells) that represent putative enhancer elements with enrichment for H3K27ac (Fig. 1g, Supplementary Fig. 1g). Alternatively, default unmethylated sites, such as CpG islands (CGIs) associated with transcription start sites, remained generally lowly methylated (Fig. 1g). A smaller subset of CGIs that were methylated in wild-type ES cells (mean >0.8, *n* = 2160/21,012) showed a strong gain to 0.56 mean methylation in DKO^dCas9–cat3a^ cells after 7 days, which may be linked to the lack of H3K4me3 (Fig. 1h, left). Supporting this, CGIs that were unmethylated in wild-type ES cells and remained so in the induced cells (*n* = 13,525, mean methylation = 0.06 and 0.09 for wild-type and 7 day-induced DKO^dCas9–cat3a^, respectively) showed greater enrichment for H3K4me3. Nonetheless, a subset of hypomethylated CGIs gained a small, but significant (*p* < 0.01) amount of DNA methylation (Fig. 1h, right, Supplementary Fig. 1h, i). Finally, when we introduced the dCas9-cat3a into normally methylated, wild-type ES cells we also observed overall protection of hypomethylated CGIs (Supplementary Fig. 1j) with little overall increase due to the already high level of genomic methylation (Supplementary Fig. 1k). These results are in agreement with the previously noted association of methylated histone H3K4 tails with protection of CGIs from the de novo DNA methylation machinery^23,24^, which we extend here to cells with ectopic methyltransferase activity.

### Ubiquitous nuclear activity is unaffected by sgRNA presence

**Fig. 2:**
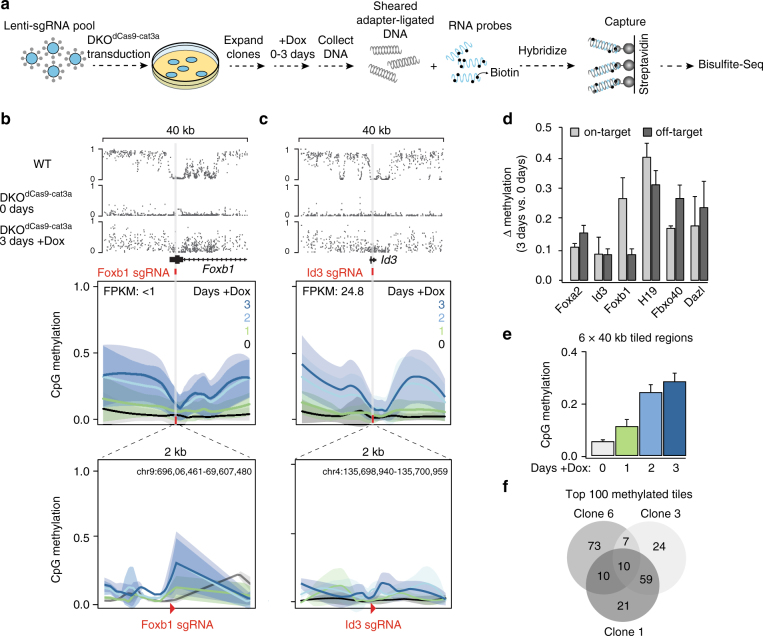
Targeted bisulfite sequencing surrounding multiple sgRNA targets. **a** Schematic describing the experimental design for the transduction of DKO^dCas9-cat3a^ cells with multiple sgRNA constructs. Cells were collected at 0, 1, 2 and 3 days after Dox treatment. Extracted DNA was hybridized to capture probes for enrichment of target loci. See Methods section for more details. **b** Top: tracks displaying methylation levels for wild-type (WT) ES cells and DKO^dCas9-cat3a^ at 0 and 3 days of Dox treatment. The target site for the Foxb1 sgRNA is displayed with 20 kb on either side, captured by our custom probes. Below: loess-smoothed methylation levels for 0, 1, 2 and 3 days of Dox treatment for the full region shown at the top, as well as a zoomed in region displaying the 2 kb surrounding the targeted locus. The sgRNA position is indicated as a red bar in the top part and red arrowhead in the bottom zoom in. **c** Same as in **b**, but for the *Id3* locus. **d** The difference in methylation after 3 days of dCas9-cat3a expression for the on-target locus (200 bp downstream of the sgRNA) as well as all other captured 200 bp tiles with mean methylation level +/- 0.05 of the on-target locus in WT ES cells (collectively termed “off-target”). Error bars display standard deviation for three clones. **e** Mean methylation for all CpGs within six 40 kb target regions that are covered in three independent clones over the Dox time course. Error bars display standard deviation for the three clones. **f** Venn diagram showing the overlap between the top 100 most highly methylated 200 bp tiles for the three independent clones

To explore if the addition of one or more sgRNAs would constrain the ubiquitous nuclear activity of the dCas9-cat3a to selected target loci, we stably integrated sgRNAs into our DKO^dCas9–cat3a^ cell line and compared on- and off-target methylation to our no guide controls. First, we tested one sgRNA against the *Dazl* promoter, a highly methylated, lowly expressed locus in wild-type ES cells with reduced CpG methylation in our DKO^dCas9–cat3a^ cell line. We independently validated the targeting efficiency of this sgRNA through the transient expression of an active Cas9 enzyme, and observed efficient cutting that mapped to the expected target site (Supplementary Fig. 2a). Interestingly, the addition of the Dazl sgRNA prior to Dox induction did not prevent the global gain of CpG methylation when compared to the no guide condition (Supplementary Fig. 2b). Moreover, while we observed a gain of CpG methylation around the sgRNA target sequence, this increase also occurred independently of the sgRNA, making it difficult to quantify the actual on-target activity for this locus (Supplementary Fig. 2c). To explore the possibility of multi-loci targeting and to see whether additional guides would help reduce the off-target activity, we next transduced our DKO^dCas9–cat3a^ cell line with a pool of ten sgRNAs that target representative loci with distinct methylation and expression levels in wild-type ES cells (Fig. 2a, Supplementary Fig. 3a). We used universal primers for the backbone to confirm the presence of sgRNAs via amplicon sequencing in several clonal lines (see Methods section) and initially focused on clone 3 in which eight of the ten sgRNAs were integrated. To assess methylation at our regions of interest with high coverage, we designed a specific set of 120 bp hybrid capture probes for targeted bisulfite sequencing, tiling 20 kb on either side of the sgRNA target. This strategy provides high-resolution information of the target locus and a representative bidirectional genomic sampling surrounding it (Fig. 2a; Supplementary Fig. 3b, c; Supplementary Table 1). Given the rapid rate of methylation gain initially observed, we chose earlier time points and collected samples 1, 2 and 3 days post Dox treatment as well as an uninduced (0 days) control (Fig. 2a–c). Similar to our prior observations, off-target methylation accumulated along each captured region (Fig. 2b, c; Supplementary Fig. 3c), demonstrating that even multiple sgRNAs are not sufficient to prevent dCas9-cat3a from acting off-target. Of interest, we did observe on-target methylation at the *Foxb1* locus, which is unmethylated and not expressed in wild-type ES cells, while other sites such as the also unmethylated but highly expressed *Id3* locus did not accumulate target methylation above background (Fig. 2c, d; Supplementary Fig. 3c). It therefore appears that the on-target activity is somewhat context dependent and the expression status may have some impact on how susceptible a locus is to methylation gain in response to dCas9–cat3a recruitment. Overall, on-target vs. off-target methylation accumulated faster at only two out of six loci (Fig. 2d). This was consistent across three independent clones which showed a reproducible global gain of methylation across all captured tiles (Fig. 2e). Notably, although mean off-target methylation was comparable for all CpGs, we found variability in the affected regions between the clones (Fig. 2f), which may suggest a largely random off-target activity at accessible sites. Taken together, these results show that the presence of sgRNAs can increase on-target methylation, but has no detectable impact on reducing global dCas9-cat3a off-target activity.

### Catalytic domain of Dnmt3a alone exhibits similar activity

Given these results, it was important to determine whether off-target methylation was mediated by dCas9 scanning the DNA independently of an sgRNA^25^ or related to the unspecific activity and ubiquitous nuclear presence of the catalytic domain of Dnmt3a (cat3a) itself. To explore this we generated two additional inducible cell lines, one expressing only the Dnmt3a catalytic domain (DKO^cat3a^) and a second one expressing the full-length Dnmt3a (DKO^Dnmt3a^, that includes both cat3a and its N-terminal regulatory domain; Supplementary Fig. 4a). After around two weeks of induction, DKO^Dnmt3a^ cells showed near wild-type levels of CpG methylation, while DKO^cat3a^ and DKO^dCas9–cat3a^ cells reached only intermediate levels (Supplementary Fig. 4b, c). Although mechanistic details remain to be elucidated, the different remethylation behaviors may result from reduced de novo activity and/or less efficient inheritance in the absence of the N-terminal regulatory domain of Dnmt3a. Furthermore, despite the fact that in principle any cytosine is a potential target for de novo methylation, we found that only cytosines in the CpG context were methylated in DKO^dCas9–cat3a^ and DKO^cat3a^ cells (Supplementary Fig. 4d, e). In contrast, the DKO^Dnmt3a^ cells showed methylation of cytosines in both the CpG and CpA context, the latter being the most frequently methylated non-CpG dinucleotide^26^. This suggests a possible dependence on the Dnmt3a regulatory domain for the acquisition of non-CpG methylation.

### dCas9-cat3a induces off-target methylation in somatic cells

**Fig. 3:**
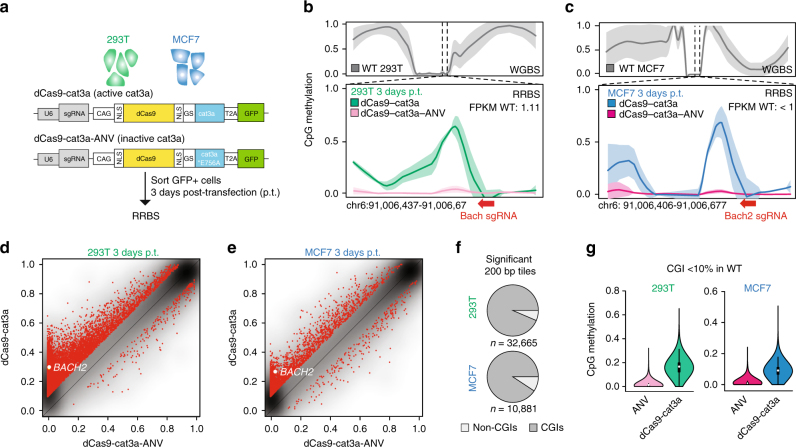
Global off-target CpG methylation after transient dCas9-cat3a transfection. **a** 293T human embryonic kidney and MCF7 human breast cancer cells were transfected with pdCas9-DNMT3A-EGFP (Addgene #71666) or pdCas9-DNMT3A-EGFP (ANV) (Addgene #71685) with an sgRNA targeting *BACH2*. Cells were collected 3 days post-transfection (p.t.) and RRBS was performed. **b** Top: loess-smoothed CpG methylation for 293T cells (WGBS) highlighting the location of the *BACH2* target within a CpG island. Bottom: the on-target smoothed methylation levels (RRBS) for 293T cells transfected with dCas9-cat3a or dCas9-cat3a-ANV 3 days p.t. The bold line indicates the mean methylation level with standard deviation shown by shaded fill. The position and orientation of the Bach2 sgRNA is indicated with a red arrow. **c** same as in **b** but for MCF7 cells. **d** Smooth scatter plot displaying mean methylation over 200 bp tiles in dCas9-cat3a and dCas9-cat3a-ANV transfected 293T cells 3 days p.t. with the Bach2 sgRNA (total tiles: 219,949). Red dots indicate tiles with significant changes in methylation (adj. *p*-value < 0.05 (*t*-test) and difference >10%, significant tiles: 32,665). The *BACH2* locus is significant and highlighted as a white dot. **e** Same as in **d** but for MCF7 cells (total tiles: 200,157; significant tiles: 10,881). **f** The proportion of significantly differentially methylated 200 bp tiles that are located within CpG islands (CGIs) for 293T and MCF7 cells. **g** Methylation levels for CGIs that have <10% methylation in WT cells shown for dCas9–cat3a–ANV and dCas9–cat3a transfected 293T/MCF7 cells

While our inducible ES cell lines have the advantage of homogenous induction and temporal control, most prior studies have selected transient transfection as the method of choice for introducing dCas9 epigenetic effectors. Therefore, we decided to compare the consequences of a transient transfection strategy^13^ in our methylation depleted, maintenance competent DKO^zero^ system (Supplementary Fig. 5a) as well as in two frequently utilized human somatic cell lines (293T; human embryonic kidney and MCF7; human breast adenocarcinoma, Fig. 3a). Consistent with the results of the inducible line, we found a global gain of CpG methylation (and lack of non-CpG methylation) when transiently transfecting our DKO^zero^ line (Supplementary Fig. 5a–c). Importantly, no gain of methylation was observed when the cells were transfected with a control construct encoding a mutated (E756A), catalytically inactive version of cat3a (dCas9-cat3a-ANV^13^, Supplementary Fig. 5d, e). For the 293T and MCF7 cells we utilized a previously described *BACH2* sgRNA and observe on-target methylation that is comparable to their published results^13^ (Fig. 3b, c). Of note, *BACH2* is either not or only marginally expressed in both of our somatic cell lines (FPKM 293T: 1.11, FPKM MCF7: < 1). This low expression may render the locus more amenable to targeted methylation, similar to the *Foxb1* locus in the mouse ES cells (Fig. 2b). Despite successful on-target methylation, we observed an extensive accumulation at off-target sites (Fig. 3d, e). Notably, both cell lines are globally methylated—unlike our DKO^zero^ line—and therefore make it more difficult to determine the exact scale of the off-target activity. When we compared 200 bp tiles between the dCas9-cat3a-ANV control and the dCas9-cat3a, 91% (MCF7) and 94% (293T) of the significantly different tiles (adj. *p*-value <0.05 (*t*-test) and difference in methylation >0.1) overlapped with otherwise lowly methylated CGIs (Fig. 3d–f), with targets varying between 293T and MCF7 cells (Supplementary Fig. 5f). Biological replicates showed a similar behavior, with 50% (293T Rep1) and 70% (293T Rep2) significant tiles being shared targets, indicating that dCas9-cat3a is acting genome-wide with no obvious sequence preference (Supplementary Fig. 5g). To more closely inspect the gain of methylation, we performed read-level analysis and find that for both on- and off-target sites only half of the reads show any methylation (Supplementary Fig. 5h, i), suggesting either a mixed population of cells or that the de novo methylation is added to only one strand generating hemimethylated DNA. In agreement with previous reports we observed a decrease in the *BACH2* target methylation within a few days post-transfection (Supplementary Fig. 5j)^13,27^. Similarly, most off-target methylation decreased within 7 days post-transfection (Supplementary Fig. 5k), which is somewhat surprising given the low expression levels of TET enzymes (Supplementary Fig. 5l). This makes an active mechanism less likely and raises important questions regarding the stability of DNA methylation when it is added out of its normal context^2^.

### dCas9-cat3a activity has limited impact on gene expression

To further investigate what renders loci more or less susceptible to off-target methylation, and considering that the majority of hypermethylated CGIs were located in promoter regions (Supplementary Fig. 6a), we wanted to explore how gained promoter methylation is linked to associated gene expression. We collected matched RNA samples from our 293T cells before and after transfection and performed RNA-sequencing which confirmed the high abundance of cat3a transcripts that should originate from the exogenous dCas9–cat3a, stable levels of DNMT1 and background levels of DNMT3B (Supplementary Fig. 6b). Importantly, the dCas9–cat3a complex predominantly added methylation to promoters of either silenced or lowly expressed genes (Supplementary Fig. 6c, d) and was only retained at a subset of lowly transcribed genes after 7 days post-transfection (Supplementary Fig. 6c, Supplementary Data 1). Despite the gain of methylation, the vast majority of hypermethylated targets were not associated with significant changes in gene expression in the steady state (Supplementary Fig. 6e). In line with the fact that DNA methylation rarely acts as a primary silencing mechanism, our results suggest that silencing actively expressed genes using dCas9-cat3a is not trivial and may require recruitment of additional repressive effectors^10,28^.

## Discussion

Since the emergence of the CRISPR-Cas9 system for genome editing, it has evolved into a versatile toolbox for numerous applications, including the targeted manipulation of the epigenome using dCas9-coupled epigenetic effector proteins. Initial proof-of-concept studies have highlighted the general applicability of the system, thereby raising the possibility for functional dissection of individual epigenetic features and their direct impact on gene regulation^8^. However, this requires precise activity and to date a careful evaluation of the global on- vs. off-target activity has not been reported. Our study offers a unique tool (the DKO^zero^ line) to track all dCas9-methyltransferase activity that can be cleanly separated from any endogenous de novo methylation by Dnmt3a and Dnmt3b and therefore provides a first true account of the unexpected genome-wide DNA methylation footprints left by dCas9-coupled to the catalytic domain of Dnmt3a. We found that wild-type ES cells appear less susceptible to CGI off-target methylation than their somatic counterparts, which may in part be explained by the more frequent enrichment of H3K4 methylation at CGIs in pluripotent cells and also the higher levels of Tet expression (Supplementary Fig. 5l)^29,30^. Importantly, while sgRNAs can direct CpG methylation to targets in certain contexts, global off-target activity appears largely unaffected by the sgRNA presence, occurs regardless of experimental-setup (transfection vs. transduction) and across multiple cell types. This extensive off-target activity is perhaps not surprising considering that the amount of free nuclear dCas9-cat3a is much higher than the target bound complex as each cell has only two alleles for a single copy locus that match the sgRNA. In the future, it would be interesting to explore whether redirecting the complex to inert genomic loci, such as abundant silent repeat elements or other ectopic targets would have an impact on reducing off-target activity by skewing its nuclear activity sufficiently to maximize the on- to off-target ratio. Furthermore, it remains to be seen whether other epigenetic effector fusions show similar global effects, although regulators of de novo DNA methylation may be particularly prone, as even transient interactions result in the addition of methyl groups that can subsequently be mitotically inherited through ubiquitously expressed DNMT1. Although we and others also highlight the transient nature of transfection-based methylation gain^10,13^, which will require further studies to delineate the underlying mechanism of failed, incomplete maintenance or active clearance. Nonetheless, sustained dCas9-cat3a activity may result in more permanent changes and it is worth considering the possibility that in some cases the aberrantly methylated loci could create a selective advantage that results in an undesired clonal expansion. Moreover, while we observed only limited effects on gene expression, we envision that these may be more striking in a system where cells are induced to change identity and have to activate previously silent regulators. In summary, our findings and the possible implications highlight the need to further investigate these valuable tools as well as remind us to consider the ubiquitous activity of dCas9-fused epigenetic effector proteins in experimental designs and interpretations.

## Methods

### dCas9-cat3a plasmid design and construction

We modified the dCas9 sequence of the pcDNA-dCas9 plasmid (Addgene #47106) to include a BsaI restriction site followed by EcoRI and a Kozak sequence (N-terminus), as well as a BsiWI restriction site, P2A-GFP, followed by EcoRI and BsaI restriction sites (C-terminus). The C-terminal Nuclear Localization Sequence (NLS) was removed, the N-terminal NLS was left intact. Following BsaI digest, the dCas9-BsiWi-P2A-GFP fragment was subcloned into the FUW backbone (Addgene #20723). The BsiWI restriction site was used to clone in the catalytic domain of Dnmt3a (cat3a, last 301 amino acids of Dnmt3a Isoform 1), with addition of a two amino acid linker (GS) between dCas9 and cat3a.

### LentiGuide-Puro cloning and transduction

The LentiGuide-Puro plasmid (Addgene #52963) was used for downstream sgRNA integration. sgRNAs were subcloned using Gibson Assembly (see also: Research Gate DOI: 10.13140/RG.2.1.1941.4161). Lentiviral particles were produced and the reset DKO^dCas9-cat3a^ line was transduced with either a single sgRNA (Dazl) or a pool of 10 sgRNAs (Dazl, Dazl_2, Foxb1, FoxA2, H19, Fbxo40, Id3, Prdm14, Dppa3, and Pou5f1) for the hybrid capture experiment.

### sgRNA sequences and coordinates

Mouse

Dazl: 5′-GAGGCGTGGGCTGCGCGCCC-3′ (mm9, chr17: 50432925–50432944)

Dazl_2: 5′-GACGGGGCAGCTACGTGAGG-3′ (mm9, chr17: 50432789–50432808)

Foxb1: 5′-GAAGGTAGAATGGGCAAGTC-3′ (mm9, chr9: 69606961–69606980)

FoxA2: 5′-GTTTTAGTTACGAAATGCTT-3′ (mm9, chr2:147872854–147872873)

H19: 5′-GGAGACTGGGTGACCACGAG-3′ (mm9, chr7: 149762698–149762717)

Fbxo40: 5′-GTACATCCCAAGTAGGCTAG-3′ (mm9, chr16: 36969271–36969290)

Id3: 5′-GCGCCTGCGGGAACTGGTGC-3′ (mm9, chr4: 135699940–135699959)

Prdm14: 5′-GAAGAATATGGATCCGGAGG-3′ (mm9, chr1: 13108873–13108892)

Dppa3: 5′-GGACAGATCCTGAGGGCTCA-3′ (mm9, chr6: 122574622–122574641)

Pou5f1: 5′-GTGTCTTCCAGACGGAGGTT-3′ (mm9: chr17: 35642849–35642868)

Human

Bach2: 5′-GAATGTAGCGATTGAGAGTGT-3′ (hg19, chr6:91006602–91006621)

### Confirming gRNA targeting efficiency

To confirm the gRNA integration and targeting efficiency we nucleofected cells with pSpCas9(BB)-2A-GFP (Addgene #PX458) and sorted for GFP 3 days post-nucleofection. Individual clones were picked and the targeting locus (*Dazl*) was amplified followed by sequencing.

### Hybrid capture and Amplicon-Seq

The reset DKO^dCas9–cat3a^ line was transduced with a pool a 10 sgRNAs (see also section on sgRNA cloning, sequences and coordinates). Upon Puromycin selection (2 μg/ml), several colonies were picked, individually propagated and then induced for 0 h, 1 day, 2 days and 3 days with doxycycline (2 μg/ml). Genomic DNA was isolated, followed by Amplicon-Seq to confirm the sgRNA composition in each clone. To do so, a fragment spanning the sgRNA integration site within the LentiGuide plasmid was amplified from the gDNA of each clonal line (FP: 5′-CGTGACGTAGAAAGTAATAATTTCTTG-3′, RP: 5′-GTTATCAACTTGAAAAAGTGGCACCG-3′). Amplicons were repaired using the End-It DNA End-Repair Kit (Epicentre), extended using Klenow fragment (3′-5′ exo) (NEB), and ligated to sequencing adapter oligos. Each library was then PCR-amplified using PFU Ultra II Hotstart Master Mix (Agilent), followed by pooling and sequencing on the MiSeq System (Illumina). sgRNAs were considered integrated if positive read counts were observed in the Amplicon-Seq.

Three clones with the following sgRNAs integrated were chosen for subsequent hybrid capture-based targeted bisulfite sequencing (custom Agilent SureSelectXT Methyl-Seq kit):

Clone 1: Dazl, Dazl_2, Foxb1, Foxa2, H19, Fbxo40, Id3, and Dppa3

Clone 3: Dazl, Dazl_2, Foxb1, Foxa2, H19, Fbxo40, Id3, and Dppa3

Clone 6: Dazl, Dazl_2, Foxb1, Foxa2, H19, Fbxo40, Id3, Dppa3, and Prdm14

On-target sites were tiled 20 kb upstream and 20 kb downstream of the sgRNA sequence using the SureDesign Custom Design Tool (Agilent). Our capture set also includes several additional control regions, which were not considered in our downstream analysis. The genomic DNA was sonicated (Covaris), ligated to methylated adapters and hybrid-selected using the designed custom 0.5-2.9 Mb SureSelect bait library (Covaris). All captured genomic DNA was bisulfited converted, PCR amplified and sequenced on the Illumina HiSeq 2500.

### Cell culture

ES cells were cultured in regular media containing 15% FBS, 1% PEN/STREP, 1% glutamine, 1% NEAA, and 10^5^ U LIF. For ES cell maintenance, dishes were coated with 0.2% gelatin and irradiated DR4 mouse embryonic fibroblasts (MEFs) were plated as a confluent layer of feeder cells. ES cells were seeded in a density of 50,000 cells per well of a six-well plate and were split every 3–4 days. Doxycycline and Puromycin were used at a concentration of 2 μg/ml. MCF7 cells (ATCC HTB-22) were grown in DMEM F-12 with 10% FBS and 1% Penicillin-Streptomycin. 293Ts (ATCC CRL-3216) were grown in DMEM supplemented with 10% FBS, 1% glutamine and 1% Penicillin-Streptomycin.

### DKO^zero^ cell line generation

For the generation of our DKO^zero^ line, we transduced the original DKO line^19^ with a previously described Dnmt1 shRNA cassette (termed TKO-like cells)^20,31^. After several passages the TKO-like cells expressing the shRNA was transiently transduced with Cre recombinase (Addgene #24593) to remove the shRNA-GFP construct, followed by sorting for GFP negative cells. Several clones were picked and propagated individually. To further enable any downstream Puromycin selection, the DKO^zero^ line was modified by disrupting its existing^19^ Puromycin resistance cassette (sgRNA: 5′-GAACTCTTCCTCACGCGCGT-3′). Individual clones were picked and DKO^zero^ clone 15.1 was chosen for all further experiments.

### DKO^dCas9-cat3a^ cell line generation

For the generation of the inducible DKO^dCas9-cat3a^ line, 20,000 DKO^zero^ ES cells were transduced with FUW-dCas9-cat3a and FUW-M2rtTA (Addgene #20342) and treated with doxycycline to allow GFP based FACS and isolation of individual GFP-positive colonies. Cells were taken off doxycycline for one passage prior to LentiGuide-Puro (Addgene #52963) transduction, (integration of Dazl sgRNA, sequence: 5′-GAGGCGTGGGCTGCGCGCCC-3′). Upon transduction, cells were selected with Puromycin for one passage, after which dCas9-cat3a was induced for two passages followed by genomic DNA extraction and whole-genome bisulfite sequencing (WGBS). To reset the DKO^dCas9-cat3a^ line, cells were passaged in 2i/Vitamin C^22^ and Dnmt1 was transiently knocked down using the shRNA described above. For the time course experiment cells were induced for 0, 2, or 7 days, followed by DNA extraction and WGBS. Homogenous induction was confirmed by inducing cells for three days with or without doxycycline, followed by FACS analysis.

### DKO^cat3a^, DKO^Dnmt3a^ and WT^dCas9-cat3a^ generation

The full-length Dnmt3a Isoform 1 or its catalytic domain (the last 301 amino acids) were fused to P2A-GFP, followed by subcloning into the FUW backbone (Addgene #20723). DKO^zero^ cells were transduced with FUW-M2rtTA (Addgene #20342) and FUW–Dnmt3a–GFP or FUW–cat3a–GFP, respectively and treated with doxycycline for about two weeks to allow GFP based FACS and isolation of individual GFP-positive colonies. For the generation of the inducible dCas9-cat3a wild-type line, V6.5 wild-type ES cells were transduced with FUW-M2rtTA and FUW–dCas9–cat3a, selected and induced as described above. Each cell line was induced with doxycycline for two passages (7 days) prior to DNA extraction and bisulfite sequencing. For the WT^dCas9–cat3a^ line, a non-induced (no doxycycline) sample was also collected for comparison.

### Transfection experiments

MCF7 and 293T cells were transfected with pdCas9–DNMT3A–EGFP (Addgene #71666) or pdCas9–DNMT3A–EGFP–ANV (Addgene #71685) including the Bach2 sgRNA (sequence: 5′-AATGTAGCGATTGAGAGTGT-3′, see also ref. 13) using FUGENE HD Transfection Reagent (Promega). Cells were sorted for GFP 3 days post-transfection if transfection efficiency was below 90% (based on visual inspection). DKO^zero^ cells were transfected with pdCas9–DNMT3A–EGFP (Addgene #71666) or pdCas9–DNMT3A–EGFP–ANV (Addgene #71685) including the Dazl sgRNA (sequence: 5′-GAGGCGTGGGCTGCGCGCCC-3′) using Xfect mouse ES cell Transfection reagent (Clontech). 293T cells were partially replated 3 days post-transfection for later collection time points at day 7 and day 15. Upon DNA extraction, the DNA was digested with DpnI for 2 h at 37 °C to digest all residual plasmid DNA. This was followed by a 0.4 × AMPure XP bead (Beckman Coulter) cleanup to select for high molecular/non-digested DNA.

### RNA-sequencing

RNA-seq libraries were prepared using the TrueSeq RNA Sample Prep v2 HS Protocol (Illumina), followed by 50 bp paired-end sequencing on the NextSeq 500 Sequencing System (Illumina).

### Reduced representation bisulfite sequencing

Genomic DNA was quantified using a Qubit 2.0 Fluorometer, and quality-assessed on an Agilent 2200 Tape-Station D1000 ScreenTape. RRBS was performed on 20 ng of each sample using the NuGen Ovation® RRBS Methyl-Seq System following the manufacturer’s recommendations except that barcoded adapter-ligated samples were pooled in groups of 8 immediately prior to bisulfite conversion with the Qiagen EpiTect Fast Bisulfite Conversion kit. Library pools were purified with a 1× Agencourt RNA XP bead cleanup and sequenced on the HiSeq 2500 or NextSeq 500 Sequencing System (Illumina). Sequenced reads were aligned to the mm9 or hg19 reference genome using BSmap^32^. Custom scripts were used to determine whether cytosines were methylated or unmethylated by observing bisulfite conversion of unmethylated cytosines in comparison to the reference sequence.

### WGBS library construction and processing

100–200 ng of genomic DNA was fragmented using a Covaris S2 for 6 min according to the following program: duty cycle 5%; intensity 10; cycle per burst 200. The sheared DNA was purified using the DNA Clean and Concentrator kit from Zymo. Bisulfite conversion of DNA was then conducted using the EZ DNA Methylation-Gold kit (Zymo Research), eluting in 15 µl low TE buffer. To minimize loss during storage, bisulfite-converted DNA was immediately processed for generating WGBS libraries using the Accel-NGS Methyl-Seq DNA library kit (Swift Biosciences). All protocols were carried out according to manufacturer’s recommendations unless specified. The libraries were sequenced with 100 bp paired-end reads on an Illumina HiSeq 2500 sequencer. 10 bp were clipped from the WGBS raw sequencing reads at either end. The reads were then aligned to the mm9 build of the mouse genome using BSmap^32^. DNA methylation calling for individual CpG sites was performed using mcall from the MOABS package^33^. CpA calling was performed using mcall with setting adjusted to detect CpAs.

### Data processing and analysis

For all methylation analysis, only CpGs covered by at least 5 reads were considered (unless otherwise stated). For genomic methylation plots, loess smoothing and standard deviation calculations were derived using the msir package in R (V1.3.1) with span set to 0.4. Violin plots were created using the R package ‘vioplot’ using standard parameters. Boxplots and heatmaps were created using R.

For all genomic feature comparisons, CpGs were intersected using BedTools v2.25.0 with: CpG islands, as previously defined^34^ or exons, introns, CGI shores, short interspersed nuclear repeats (SINEs), long interspersed nuclear repeats (LINEs), long terminal repeats (LTRs) or Intra-cisternal A-type particle (IAP) elements downloaded from the UCSC Genome Browser (mm9 or hg19 depending on data).

For the hybrid capture data, the methylation bed files were intersected with the six 40 kb target regions. IGV tracks were created with all CpGs covered by at least 5 reads. To compare on-target vs. off-target methylation, we compared the methylation level of the 200 bp immediately downstream of the sgRNA sequence (on-target), to all captured 200 bp tiles with the same methylation level in WT cells (i.e., with the same potential to become methylated; off-target). The difference between 0 day and 3 day mean methylation was calculated for the three clones including mean and standard deviation. To compare the most highly methylated tiles between clones, we selected the top 100 methylated 200 bp tiles in each sample and computed the overlap, plotting venn diagrams manually.

To generate delta methylation histograms for WT vs. WT^dCas9–cat3a^ cells, we compared mean methylation over 1 kb tiles.

To compare methylation levels between transfected samples, we tiled the genome with 200 bp tiles and performed paired *t*-tests for CpGs with ≥10 reads within the tile calculating *p*-values adjusted using the Benjamini–Hochberg model. Only tiles containing at least 3 CpGs were considered. Significant tiles had adj. *p*-value <0.05 and at least 10% difference in methylation compared to WT.

To identify significant changes in CGI methylation, the same *t*-test was performed over each CGI (with ≥3 CpGs) with significant islands selected as adj. *p*-value <0.05 with no cutoff for change in methylation.

To compare CGI methylation to H3K4me3 methylation, BedTools was used to obtain read counts per CpG island. The number of reads was then divided by the length of the CGI with the top 1% values normalized to 100 and the rest of the values scaled accordingly.

To analyze individual reads, custom perl scrips were used to extract per CpG methylation levels across reads. Reads were then plotted using R for individual loci to study examples of per-read CpG methylation. Seperately, all reads located within 200 bp windows where CpGs showed significant changes in methylation were analyzed to determine whether all reads or only a subset acquired methylation. Reads were allocated into the category “none” if no methylation existed, and “some” if we detected at least one methylated CpG.

RNA-seq data was aligned using STAR aligner followed by stringtie v1.3 to the hg19 genome. FPKM values collapsed by gene were extracted and compared for two technical replicates. Gene-specific FPKM values were TMM normalized using edgeR in R. For differential analysis (log fold change), all transcripts with FPKM <1 were removed.

FPKM levels for Tet1 and Tet2 in mouse ES and human ES were taken from two previously published papers^35,36^.

### Nuclear extraction and western blotting

Approximately six million ES cells (V6.5 and reset DKO^dCas9–cat3a^) were MEF depleted and resuspended in 7 ml lysis buffer containing 25 mM HEPES pH 7.6, 5 mM MgCl_2_, 25 mM KCl, 0.05 mM EDTA, 10% Glycerol, 0.1% IGEPAL, 1 mM DTT, 1 × Roche proteinase inhibitor. The lysate was centrifuged for 5 min at 211 × *g* and the supernatant was discarded. The precipitate was resuspended in 500 µl cold buffer containing 10 mM HEPES pH7.6, 3 mM MgCl_2_, 100 mM KCl, 0.01 mM EDTA, 10% Glycerol, 1 mM DTT, 1× Roche proteinase inhibitor to wash nuclei, centrifuged 5 min at 3000 × *g* and the supernatant was discarded. The precipitate was resuspended in 100 µl RIPA buffer containing DNase and RNase, vortexed for 20 min at 4 °C, centrifuged for 15 min at  13,523 ×* g*. Supernatant containing soluble proteins was transferred to a new tube, loading buffer (NuPAGE LDS Sample Buffer 4×, Thermo Fisher Scientific) was added to 1× and denatured for 10 min at 70°C. 15 µl of the sample was run on a NuPage 4–12% Bis-Tris protein gel in 1× MOPS buffer with 10 µl of Precision Plus Protein Dual Color Standards ladder (Bio-Rad) for 1 h at 200 V. Protein was then transferred on a nitrocellulose membrane by wet electroblotting for 2 h at 340 mA. The membrane was blocked in 5% non-fat milk, cut into two sections and incubated with mouse primary antibodies anti-DNMT1 (1:2000 Active Motif #39204) and anti-Tubulin (1:10,000 DSHB #12G10) overnight, washed three times for 10 min in PBS with 0.1% (v/v) Tween and incubated with anti-mouse-HRP secondary antibody (1:10,000 Jackson Laboratory #115-035-174) for 1 h. Following three 10 min washing steps in PBS with 0.1% Tween, the protein bands were visualized using ECL reagent (GE Healthcare). Uncropped images of Fig. 1b are available in Supplementary Fig. 1.

### Data availability

All sequencing data that support the findings of this study have been deposited in the National Center for Biotechnology Information Gene Expression Omnibus (GEO) and are accessible through the GEO Series accession number GSE87757. Gene-specific FPKM values for the RNA-seq data is also available as Supplementary Data File 1. FPKM levels for Tet1 and Tet2 in mouse ES cells and human ES cells from two previously published papers^35,36^ are available from GEO under accession codes human ES cells: GSM1112834 and GSM1112837 and mouse ES cells: GSM1842768 and GSM1842769.

## Electronic supplementary material

Supplementary Information

Peer Review File

Description of Additional Supplementary Files

Supplementary Data 1
