# Supplementary material for: Genome-wide tracking of dCas9-methyltransferase footprints
Source: Nat Commun. 2018 Feb 9;9:597. doi: 10.1038/s41467-017-02708-5 (PMC5807365; doi:10.1038/s41467-017-02708-5)
Supplement: Supplementary file 1 — Supplementary Information [file 41467_2017_2708_MOESM1_ESM.pdf]

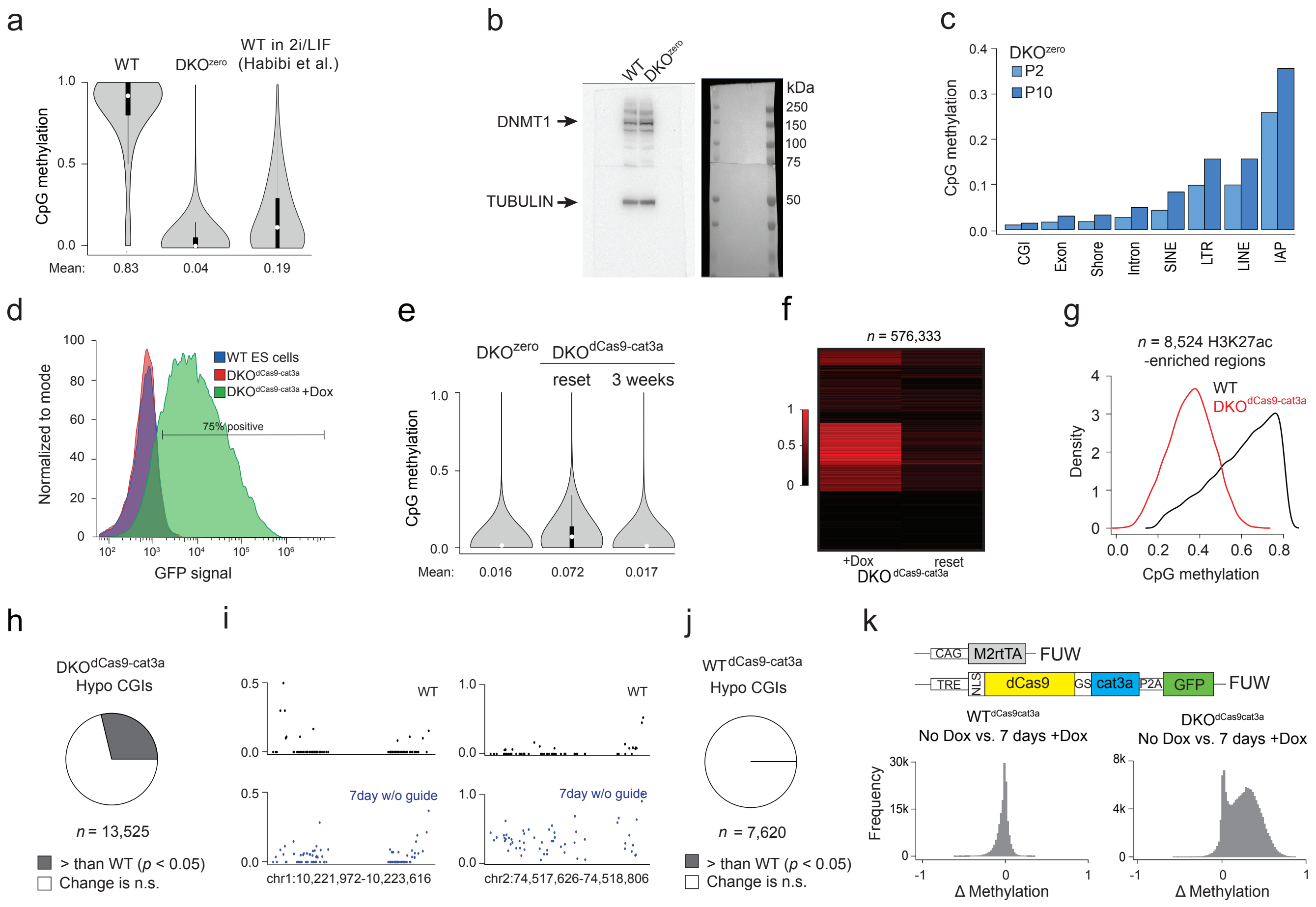

Supplementary Figure 1.

a) CpG methylation levels by WGBS of wild-type (WT) ES cells, DKO^{zero} and WT ES cells cultured in 2i/LIF (data from Habibi et al. Ref 1). **b**) Western blot of DNMT1 and TUBULIN in WT and DKO^{zero} cells. **c**) Mean methylation level by RRBS for DKO^{zero} cells at passage 2 (P2) and passage 10 (P10) for CpGs located within selected genomic features: CpG island (CGI), exon, CGI shore, intron, short interspersed nuclear element (SINE), long terminal repeat element (LTR), long interspersed nuclear element (LINE) and intracisternal A-particle element (IAP). **d**) FACS shows the population-wide induction of the dCas9-cat3a-P2A-GFP cassette after 3 days of Dox treatment. Uninduced DKO$^{dCas-cat3a}$ cells overlap with the negative control line (KH2 wild-type ES cells). **e**) Violin plots show methylation levels by RRBS (n = 764,013 CpGs) for DKO$^{dCas9-cat3a}$ after methylation reset (reset) and the same line after three weeks of passaging without Dox (3 weeks) compared to the original DKO^{zero} line. **f**) Heatmap displaying the gain of global CpG methylation (by RRBS, CpGs match **Fig. 1c**) after induction of dCas-cat3a and its global depletion (reset) upon transient culture in 2i/VitC combined with another round of transient Dnmt1 knockdown. **g**) Density plot shows methylation distribution for CpGs within regions enriched for H3K27ac (marking putative enhancer-like elements) in WT and DKO$^{dCas9-cat3a}$ cells induced for 7 days (n = 8,524). **h**) The proportion of hypomethylated CGIs (mean < 0.2 in WT) in DKO$^{dCas9-cat3a}$ that show significantly higher methylation level compared to WT (n = 13,525). n.s = non significant. **i**) Two examples of CGIs that gain methylation after DKO$^{dCas9-cat3a}$ cells were treated with Dox for 7 days. **j**) The proportion of CGIs in 7 day induced WT$^{dCas9-cat3a}$ cells that gain significant methylation compared to WT levels (n = 7,620). **k**) Top: schematic showing the experimental design: WT ES cells were transduced with the two depicted lentiviral constructs, allowing for the Dox inducible expression of dCas9-cat3a. Bottom: methylation levels over 1 kb tiles were compared in (left) WT$^{dCas9-cat3a}$ with no Dox vs 7 days on Dox or (right) DKO$^{dCas9-cat3a}$ with no Dox vs 7 days on Dox. The delta methylation histograms display the frequency of 1 kb tiles with discrete difference in methylation, a shift towards positive values indicates a gain in methylation in the WT$^{dCas9-cat3a}$ / DKO$^{dCas9-cat3a}$ in the plus Dox condition, respectively. When dCas9-cat3a was induced in WT ES cells, no positive shift was observed, whereas our DKO$^{dCas9-cat3a}$ line shows a strong positive shift (= gain of methylation) upon Dox induction.

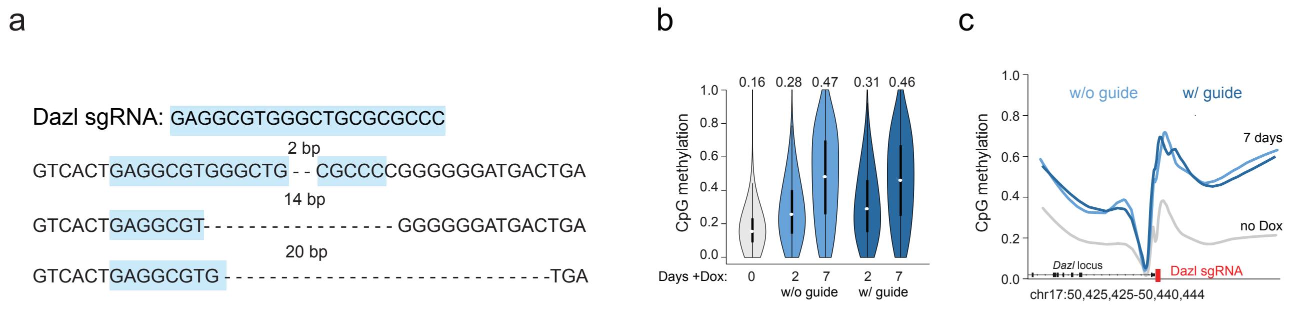

Supplementary Figure 2.

a) sgRNA validation using transient transfection of a plasmid harboring the functional Cas9 enzyme. The sgRNA sequence is highlighted in blue. Each line shows the DNA sequence within an individual clone that was cut by Cas9, dashed lines denote the deleted sequence. Three out of four colonies showed a cut at the expected target site. **b**) Methylation level by WGBS for matched CpGs in $DKO^{dCas9\text{-}cat3a}$ with 0, 2 and 7 days of Dox induction with (w/) and without (w/o) the presence of an sgRNA. White dots indicate median value, number represents mean value for each time point. **c**) Mean methylation by WGBS along the *Dazl* locus in $DKO^{dCas9\text{-}cat3a}$ after 0 or 7 days of Dox induction in presence or absence of the Dazl sgRNA. The genomic position of the matching sgRNA sequence is indicated by the red box.

a

sgRNA	Target	Methylation status in WT ES cells	Expression status in WT ES cells
1	*Foxbl*	Not methylated	Not expressed
2	*Foxa2*	Not methylated	Not expressed
3	*Id3*	Not methylated	Expressed
4	*H19*	Methylated	Expressed
5	*Dazl*	Methylated	Low expression
6	*Dazl_2*	Methylated	Low expression
7	*Fbxo40*	Methylated	Not expressed
8	*Dppa3*	Not methylated	Low expression
9	*Prdm14*	Methylated	Expressed
10	*Pou5f1*	Not methylated	Expressed

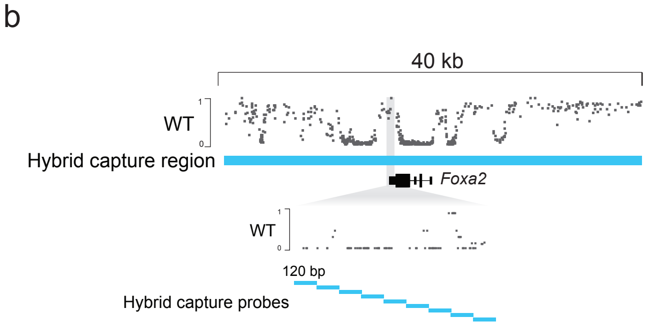

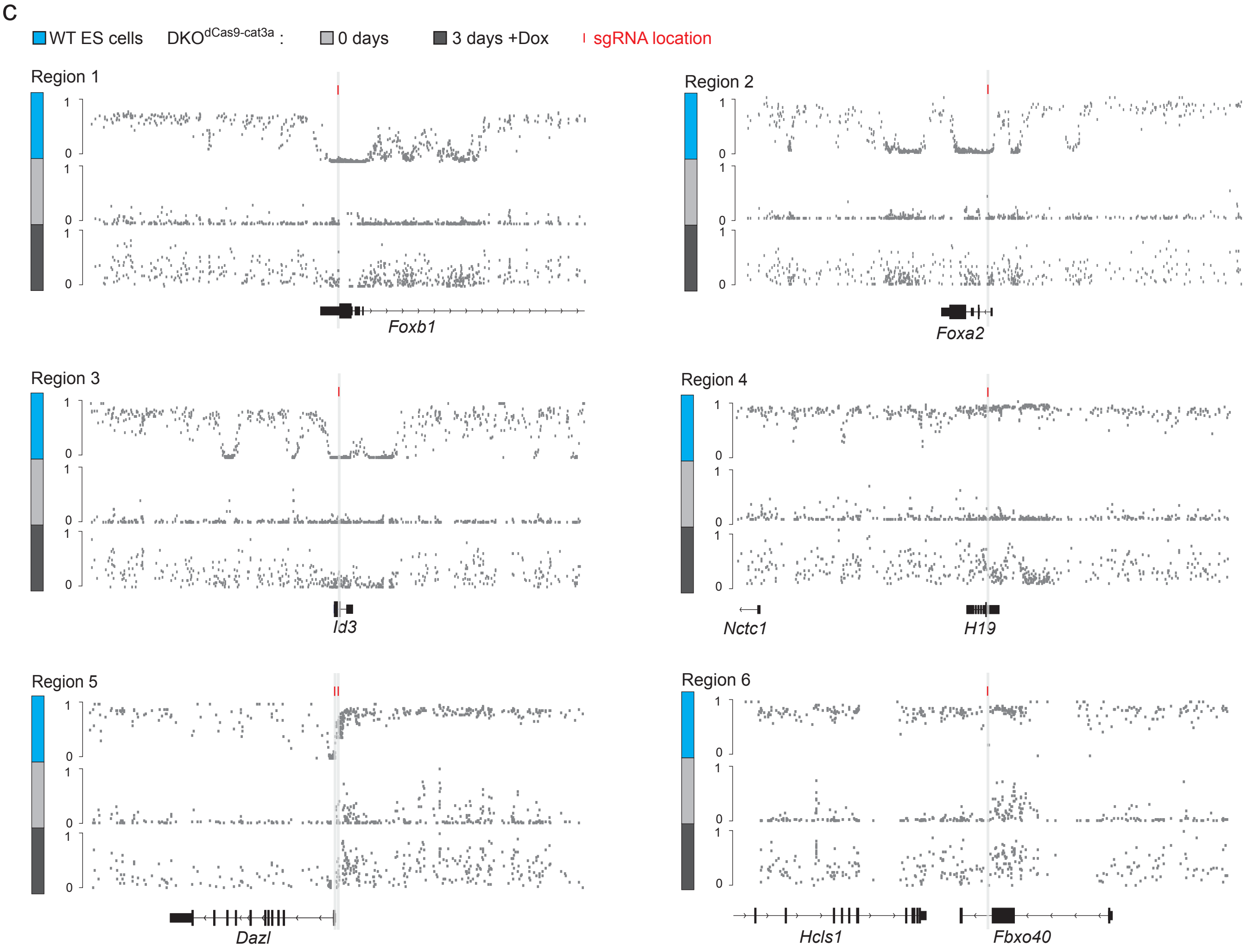

Supplementary Figure 3.
a) Summary of the target regions captured by our custom probes. See Methods section and Table S1 for more details. **b**) Schematic showing the capture probes around the *Foxa2* locus. The tiled probes are displayed in blue and demonstrate full coverage of 40 kb surrounding the sgRNA target region. **c**) Genome browser tracks for six target regions (including seven sgRNAs of the total eight that were detected in clone 3) at 5X coverage for WT ES cells, as well as clone 3 DKO$^{dCas9\text{-}cat3a}$ cells treated for 0 or 3 days with Dox. Red bars and shaded grey boxes indicate the sgRNA target.

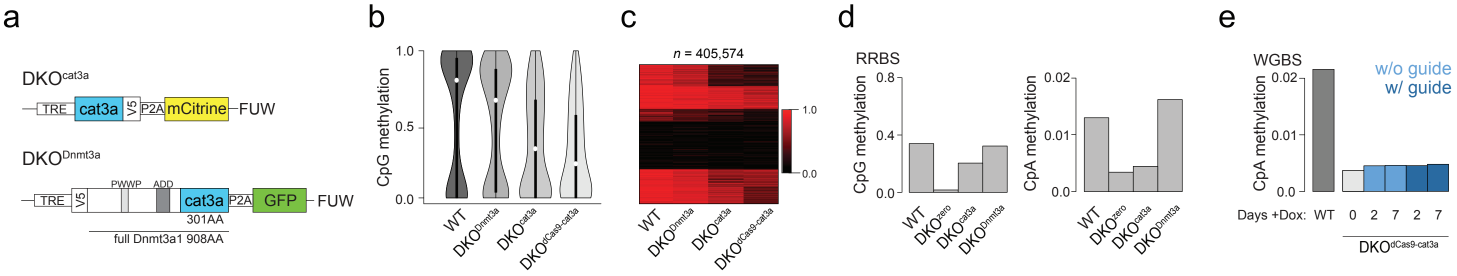

Supplementary Figure 4.

a) Simplified schematics showing the cat3a and full length Dnmt3a FUW-based lentiviral expression constructs. See Methods for more details on the plasmid backbones and cloned sequences. **b**) Methylation level by RRBS for matched CpGs in WT ES cells as well as Dox induced DKO^{Dnmt3a}, DKO^{cat3a} and $DKO^{dCas9\text{-}cat3a}$ cells (*n* = 405,574). White dots indicate median methylation values. **c**) Heatmap displays the global gain of methylation at the same CpGs as in **b** for WT ES cells, Dox induced DKO^{Dnmt3a}, DKO^{cat3a} and $DKO^{dCas9\text{-}cat3a}$ cells. **d**) Mean methylation levels for CpG (left) and CpA methylation (right) by RRBS. CpG methylation in the DKO^{Dnmt3a} line reaches almost wild-type levels while DKO^{cat3a} shows reduced CpG methylation. For non-CpG methylation, only the full length construct is able to methylate CpAs above background levels in the DKO^{zero} line. **e**) Bar plots displaying global levels of CpA methylation based on WGBS in WT ES cells and $DKO^{dCas9\text{-}cat3a}$ cells after 0, 2 and 7 days of Dox induction with (w/) or without (w/o) presence of an sgRNA.

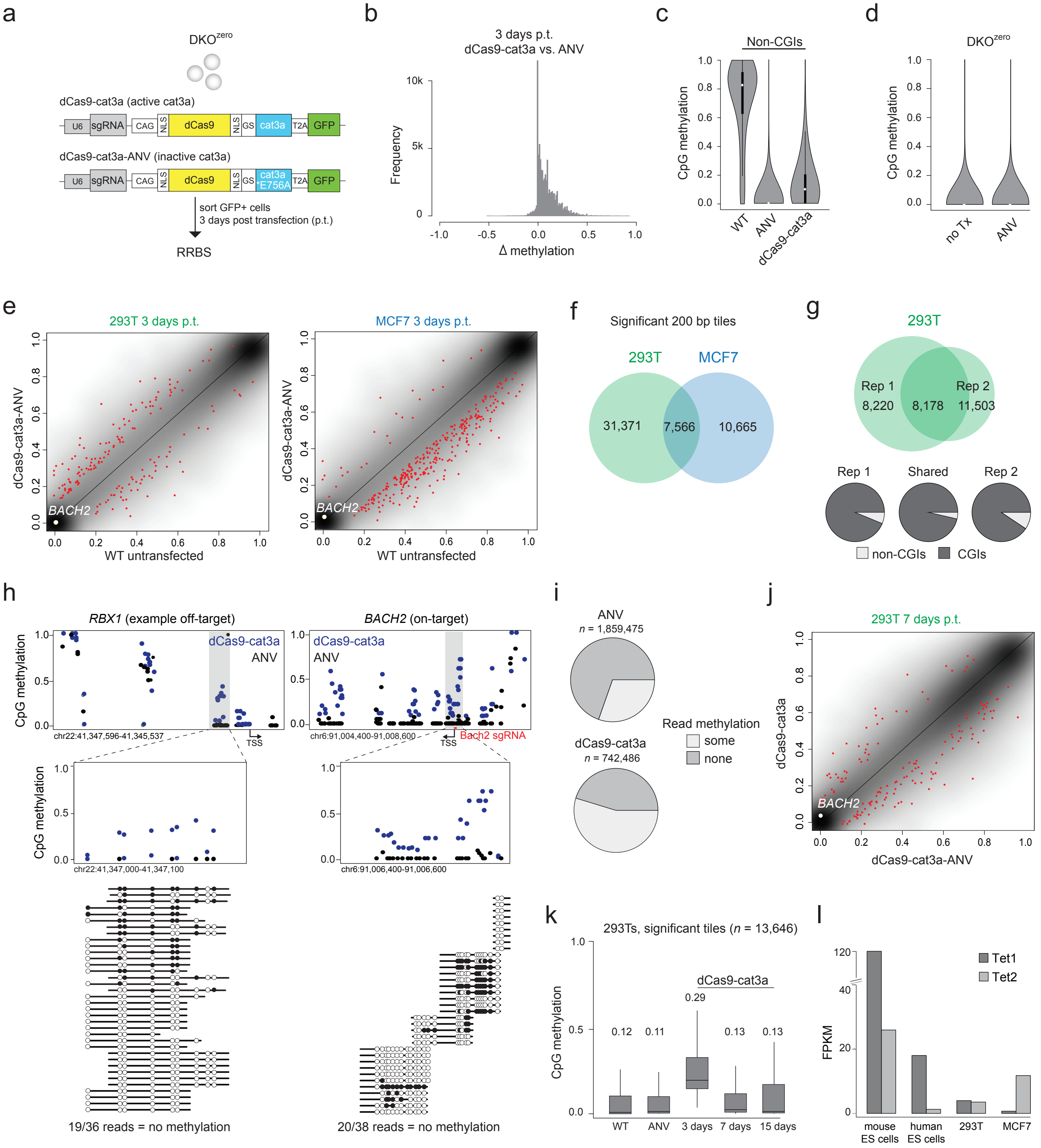

a
DKO^zero
dCas9-cat3a (active cat3a)
U6 sgRNA CAG NLS dCas9 NLS GS cat3a T2A GFP
dCas9-cat3a-ANV (inactive cat3a)
U6 sgRNA CAG NLS dCas9 NLS GS cat3a E756A T2A GFP
sort GFP+ cells
3 days post transfection (p.t.)
RRBS
b
3 days p.t.
dCas9-cat3a vs. ANV
Frequency
Δ methylation
c
Non-CGIs
CpG methylation
WT
ANV
dCas9-cat3a
d
DKO^zero
CpG methylation
no Tx
ANV
e
293T 3 days p.t.
MCF7 3 days p.t.
dCas9-cat3a-ANV
WT untransfected
BACH2
f
Significant 200 bp tiles
293T
MCF7
31,371
7,566
10,665
g
293T
Rep 1
Rep 2
8,220
8,178
11,503
Rep 1
Shared
Rep 2
non-CGIs
CGIs
h
RBX1 (example off-target)
BACH2 (on-target)
CpG methylation
dCas9-cat3a
ANV
chr22:41,347,596-41,345,537
TSS
chr6:91,004,400-91,008,600
Bach2 sgRNA
chr22:41,347,000-41,347,100
chr6:91,006,400-91,006,600
19/36 reads = no methylation
20/38 reads = no methylation
i
ANV
n = 1,859,475
dCas9-cat3a
n = 742,486
Read methylation
some
none
j
293T 7 days p.t.
dCas9-cat3a
dCas9-cat3a-ANV
BACH2
k
293Ts, significant tiles (n = 13,646)
CpG methylation
dCas9-cat3a
0.12
0.11
0.29
0.13
0.13
WT
ANV
3 days
7 days
15 days
l
FPKM
Tet1
Tet2
mouse ES cells
human ES cells
293T
MCF7

Supplementary Figure 5.

a) DKO^{zero} cells were transfected with pdCas9-DNMT3A-GFP (Addgene # 71666) or pdCas9-DNMT3A-EGFP-ANV (Addgene # 71685; ANV: inactive cat3a) with an sgRNA targeting *Dazl*. Cells were collected 3 days post-transfection (p.t.) and RRBS was performed. **b**) Histogram showing the difference in mean methylation by RRBS for 1 kb tiles where positive values represent tiles with higher methylation in the 3 days p.t. sample compared to dCas9-cat3a-ANV cells. **c**) Methylation level for non-CGIs in WT and 3 days p.t. DKO^{zero} cells. ANV: dCas9-cat3a-ANV; CGIs: CpG islands. **d**) Methylation level for DKO^{zero} cells vs. DKO^{zero} cells 3 days p.t. with dCas9-cat3a-ANV, including the Dazl sgRNA. The transfected cells show no increase in methylation. **e**) Smooth scatter plot displaying mean methylation over 200 bp tiles in WT and dCas9-cat3a-ANV (including Bach2 sgRNA) transfected 293T and MCF7 cells, 3 days p.t. Red dots indicate tiles with significant changes in methylation (adj. p-value < 0.05 and difference > 10%). **f**) Overlap of significant 200 bp tiles between MCF7 and 293T cells for all tiles covered in both samples. **g**) Number of unique and shared tiles that were significantly differentially methylated in two biological replicates of 293T cells transfected with either dCas9-cat3a-ANV or dCas9-cat3a (including Bach2 sgRNA). The proportion of targets that are located within CGIs are shown below. **h**) Mean methylation for individual CpGs along a 200 bp tile within the *RBX1* gene (off-target) and *BACH2* locus (on-target). The methylation pattern of individual sequencing reads is displayed below (circle = CpG, filled = methylated, unfilled = unmethylated). In both examples only around 50% reads contained any methylation. **i**) When extending the analysis from **h** to all significant 200 bp tiles, we found that globally, only around half of the reads contained any methylation, where "some" methylation means at least 1 CpG methylated per read. **j**) Smooth scatter plot as described in **e** for 293T cells transfected with dCas9-cat3a-ANV or dCas9-cat3a (including the Bach2 sgRNA), 7 days p.t. **k**) Boxplots displaying methylation levels for 200 bp tiles that significantly gain methylation in dCas9-cat3a transfected 293T cells 3 days p.t. Methylation levels for matched tiles are displayed for WT and dCas9-cat3a-ANV transfected cells (3 days p.t.) as well as dCas9-cat3a transfected cells (3, 7 and 15 days p.t.) Boxplots show the median as a horizontal line, surrounded by a box showing the interquartile range. Whiskers extend to the most extreme data point that is no more than 1.5 times the interquartile range. **l**) Tet1 and Tet2 expression (FPKM) for mouse ES cells, human ES cells, 293T and MCF7 cells (data for mouse ES cells from Ref 2, data for human ES cells from Ref 3).

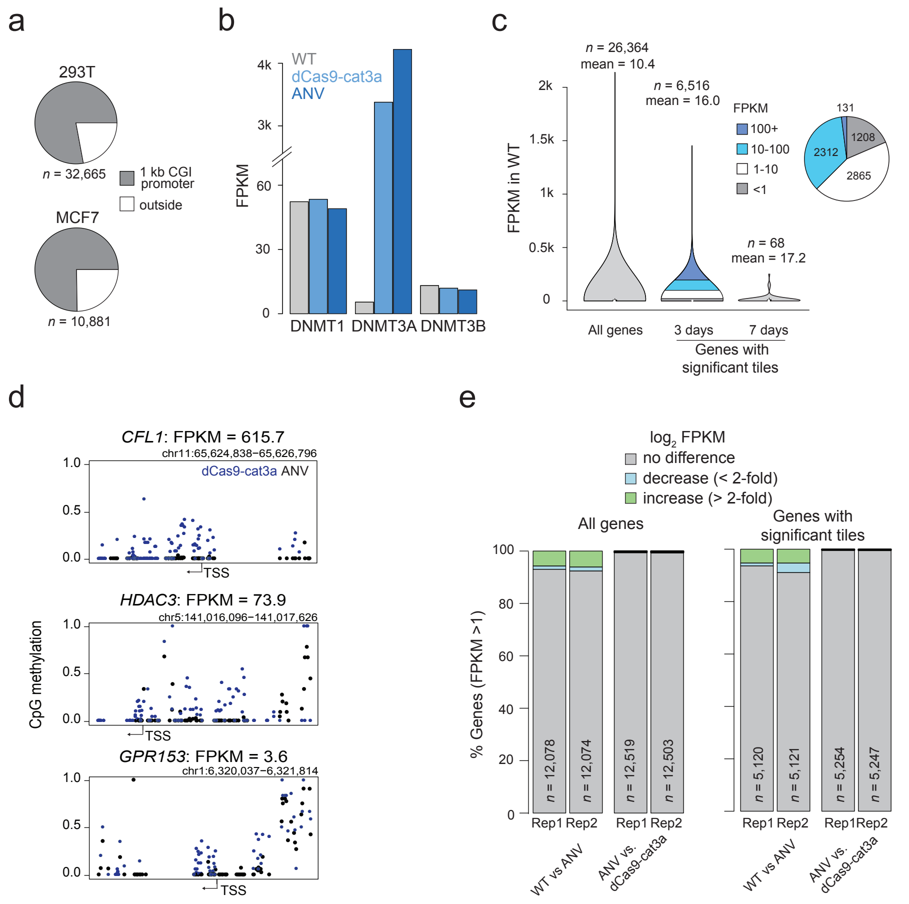

Supplementary Figure 6.

a) Pie charts showing the proportion of significantly differentially methylated 200 bp tiles in 293T and MCF7 cells that were located within 1 kb of a CGI promoter (*n* = 32,665 and 10,881, respectively). **b**) FPKM for DNMT1, DNMT3A and DNMT3B in WT, dCas9-cat3a-ANV and dCas9-cat3a transfected 293T cells 3 days p.t. The transfection results in a high increase of DNMT3A transcript counts, while DNMT1 and DNMT3B expression remain unaffected. **c**) WT expression levels (FPKM) for all genes in 293T cells and genes that contain a significant 200 bp tile within the transcript (94% within the promoter region) 3 days p.t with dCas9-cat3a and genes that contain a significant tile within the transcript 7 days p.t. The 3 day p.t. sample is divided into silent (FPKM= <1), lowly expressed (FPKM = 1-10), expressed (FPKM = 10-100) and highly expressed genes (FPKM = >100). **d**) A single copy gene from each category described in **c**, displayed with methylation levels for dCas9-cat3a-ANV and dCas9-cat3a. All three genes show a gain in methylation in presence of dCas9-cat3a. **e**) (left) Proportion of all genes and (right) genes with a significant 200 bp tiles within 1 kb of the promoter (FPKM >1) in 293T cells that display at least 2-fold increase or decrease (3 days p. t.).

					40 kb region		500 bp upstream		500 bp downstream			
sgRNA	Target	Sequence	Guide location	Region type	Mean methylation WT ES cells	*n* CpGs	Mean methylation	*n* CpGs	Mean methylation	*n* CpGs	Target Gene	FPKM in WT ES cells
1	*Foxa2*	GTTTTAGTTACGAAATGCTT	chr2:147,872,851-147,872,873	Hypomethylated, not expressed	0.284	686	0.072	17	0.652	4	*Foxa2*	<1
2	*Id3*	GCGCCTGCGGGAACTGGTGC	chr4:135,699,940-135,699,959	Hypomethylated, expressed	0.350	752	0.002	41	0.005	27	*Id3*	24.84
3	*H19*	GGAGACTGGGTGACCACGAG	chr7:149,762,698-149,762,717	Hypermethylated, expressed	0.805	480	0.835	7	0.858	11	*H19*	27.08
4	*Foxb1*	GAAGGTAGAATGGGCAAGTC	chr9:69,606,961-69,606,980	Hypomethylated, not expressed	0.364	713	0.038	18	0.025	34	*Foxb1*	<1
5	*Fbxo40*	GTACATCCCAAGTAGGCTAG	chr16:36,969,271-36,969,290	Hypermethylated, not expressed	0.740	333	0.871	7	0.866	13	*Fbxo40*	<1
6	*Dazl*	GAGGCGTGGGCTGCGCGCCC	chr17:50,432,925-50,432,944	Hypermethylated, low expression	0.677	406	0.627	39	0.803	32	*Dazl*	2.79
7	*Dazl_2*	GACGGGGCAGCTACGTGAGG	chr17:50,432,789-50,432,808	Hypermethylated, low expression	N/A	N/A	N/A	N/A	N/A	N/A	N/A	N/A
8	*Dppa3*	GGACAGATCCTGAGGGCTCA	chr6:122,574,622-122,574,641	Not covered by probes	N/A	N/A	N/A	N/A	N/A	N/A	N/A	N/A
9	*Prdm14*	GAAGAATATGGATCCGGAGG	chr1:13,108,873-13,108,892	Not integrated in all clones	N/A	N/A	N/A	N/A	N/A	N/A	N/A	N/A
10	*Pou5f1*	GTGTCTTCCAGACGGAGGTT	chr17:35,642,849-35,642,868	Not integrated in all clones	N/A	N/A	N/A	N/A	N/A	N/A	N/A	N/A

Supplementary Table 1
sgRNA targets and hybrid capture analysis
