# Supplementary material for: Genome-wide tracking of dCas9-methyltransferase footprints
Source: Nat Commun. 2018 Feb 9;9:597. doi: 10.1038/s41467-017-02708-5 (PMC5807365; doi:10.1038/s41467-017-02708-5)
Supplement: Supplementary file 3 — Description of Additional Supplementary Files [file 41467_2017_2708_MOESM3_ESM.pdf]

Description of Additional Supplementary Files

File Name: Supplementary Data 1
Description: A table listing the FPKM for every gene in WT 293T cells and 293T cells transiently transfected with dCas9-cat3a or dCas9-ANV. There are two technical replicates per sample.
