# Supplementary material for: Genome-wide tracking of dCas9-methyltransferase footprints
Source: Nat Commun. 2018 Feb 9;9:597. doi: 10.1038/s41467-017-02708-5 (PMC5807365; doi:10.1038/s41467-017-02708-5)
Supplement: Supplementary file 2 — Peer Review File [file 41467_2017_2708_MOESM2_ESM.pdf]

Editorial Note: this manuscript has been previously reviewed at another journal that is not operating a transparent peer review scheme. This document only contains reviewer comments and rebuttal letters for versions considered at Nature Communications.
